# Standard metabolic rate predicts growth trajectory of juvenile Chinese crucian carp (*Carassius auratus*) under changing food availability

**DOI:** 10.1242/bio.025452

**Published:** 2017-07-27

**Authors:** Ling-Qing Zeng, An-Jie Zhang, Shaun S. Killen, Zhen-Dong Cao, Yu-Xiang Wang, Shi-Jian Fu

**Affiliations:** 1 Laboratory of Evolutionary Physiology and Behavior, School of Life Sciences, Chongqing Normal University, Key Laboratory of Animal Biology of Chongqing, Chongqing 401331, China; 2 Institute of Biodiversity, Animal Health & Comparative Medicine, College of Medical, Veterinary & Life Sciences, University of Glasgow, Glasgow, G12 8QQ, UK; 3 Department of Biology, Queen's University, Ontario, K7L 3N6, Canada

**Keywords:** Growth, Starvation tolerance, Standard metabolic rate, Intraspecific variation, Food availability, *Carassius auratus*

## Abstract

Phenotypic traits vary greatly within populations and can have a significant influence on aspects of performance. The present study aimed to investigate the effects of individual variation in standard metabolic rate (SMR) on growth rate and tolerance to food deprivation in juvenile Chinese crucian carp (*Carassius auratus*) under varying levels of food availability. To address this issue, 19 high and 16 low SMR individuals were randomly assigned to a satiation diet for 3 weeks, whereas another 20 high and 16 low SMR individuals were assigned to a restricted diet (approximately 50% of satiation) for the same period. Then, all fish were completely food-deprived for another 3 weeks. High SMR individuals showed a higher growth rate when fed to satiation, but this advantage of SMR did not exist in food-restricted fish. This result was related to improved feeding efficiency with decreased food intake in low SMR individuals, due to their low food processing capacity and maintenance costs. High SMR individuals experienced more mass loss during food deprivation as compared to low SMR individuals. Our results here illustrate context-dependent costs and benefits of intraspecific variation in SMR whereby high SMR individuals show increased growth performance under high food availability but had a cost under stressful environments (i.e. food shortage).

## Abstract (precis)

**Summary:** Our study documented how the relationship between variation in standard metabolic rate and growth is altered by the food availability in fish.

## INTRODUCTION

Basal metabolism represents the minimum energy required to sustain life at a particular temperature, and is analogous to standard metabolic rate (SMR) in ectotherms and basal metabolic rate (BMR) in endotherms (Frappell and Butler, 2004; Van Leeuwen et al., 2012). SMR can vary by as much as two- to threefold among individuals within a population, even after controlling for factors such as body mass, temperature, sex or age (Metcalfe et al., 1995; Cutts et al., 1998; Armstrong et al., 2011). Many physiological and ecological studies have examined intra-individual variation in SMR because it is often assumed to reflect the ‘idling cost’ of a metabolic ‘engine’ used to support costly behavioral or physiological processes (Careau et al., 2008). From an evolutionary perspective, trade-offs in energy allocation may exist which could affect the fitness of individuals over their life history (Burton et al., 2011). Empirical studies suggest that a higher SMR is often positively correlated to various ecologically relevant behaviors (e.g. activity, aggression) (Metcalfe et al., 2016), meal processing rate (Millidine et al., 2009) and growth (Álvarez and Nicieza, 2005; Reid et al., 2012; Van Leeuwen et al., 2012). However, animals with a higher SMR may experience costs under stressful environments (i.e. food shortage) due to their higher maintenance metabolism (Burton et al., 2011; Killen et al., 2013).

Like many other vertebrates, many fish species experience extreme spatial and temporal variation in food availability (McCue, 2010) in response to natural factors and habitat modification by human activity (Martin-Smith and Armstrong, 2002). During periods of eutrophication during the summer, for example, Atlantic croaker (*Micropagonias undulates*) (Powers et al., 2005), or while overwintering, fishes can often go with little or no food for days or weeks at a time (Auer et al., 2016). The ability to tolerate reduced food availability (using mass-loss as an index for vulnerability) varies greatly not only among species (McCue, 2010) but also within species (Dupont-Prinet et al., 2010; Killen et al., 2011; McKenzie et al., 2014; Auer et al., 2015a,b). Therefore, there is a need to understand the ecological consequences and functional relevance of intraspecific variation in SMR, in the context of environmental change and associated fluctuations in food availability.

We studied intraspecific variation in SMR and its effects on responses to fluctuations in food availability in juvenile crucian carp (*Carassius auratus*). This is a freshwater teleost fish, which is widely distributed in the rivers, lakes and other water bodies in China. Like other fish species that occupy the same habitat, crucian carp often experience spatial and temporal fluctuations in food availability in their natural habitat (e.g. the Yangtze River) (Xu et al., 1999; Zeng et al., 2012). Our study examined the consequences of inter-individual variation in SMR for somatic growth of this Cyprinid fish under different levels of food availability. Specifically, we sought to test whether high SMR individuals would grow faster under conditions of high food availability but lose mass most quickly under conditions of low food availability. The results provide insight into how food availability and SMR interact to affect the growth performance in fish in the face of environmental changes.

## RESULTS

**Fig. 1.**
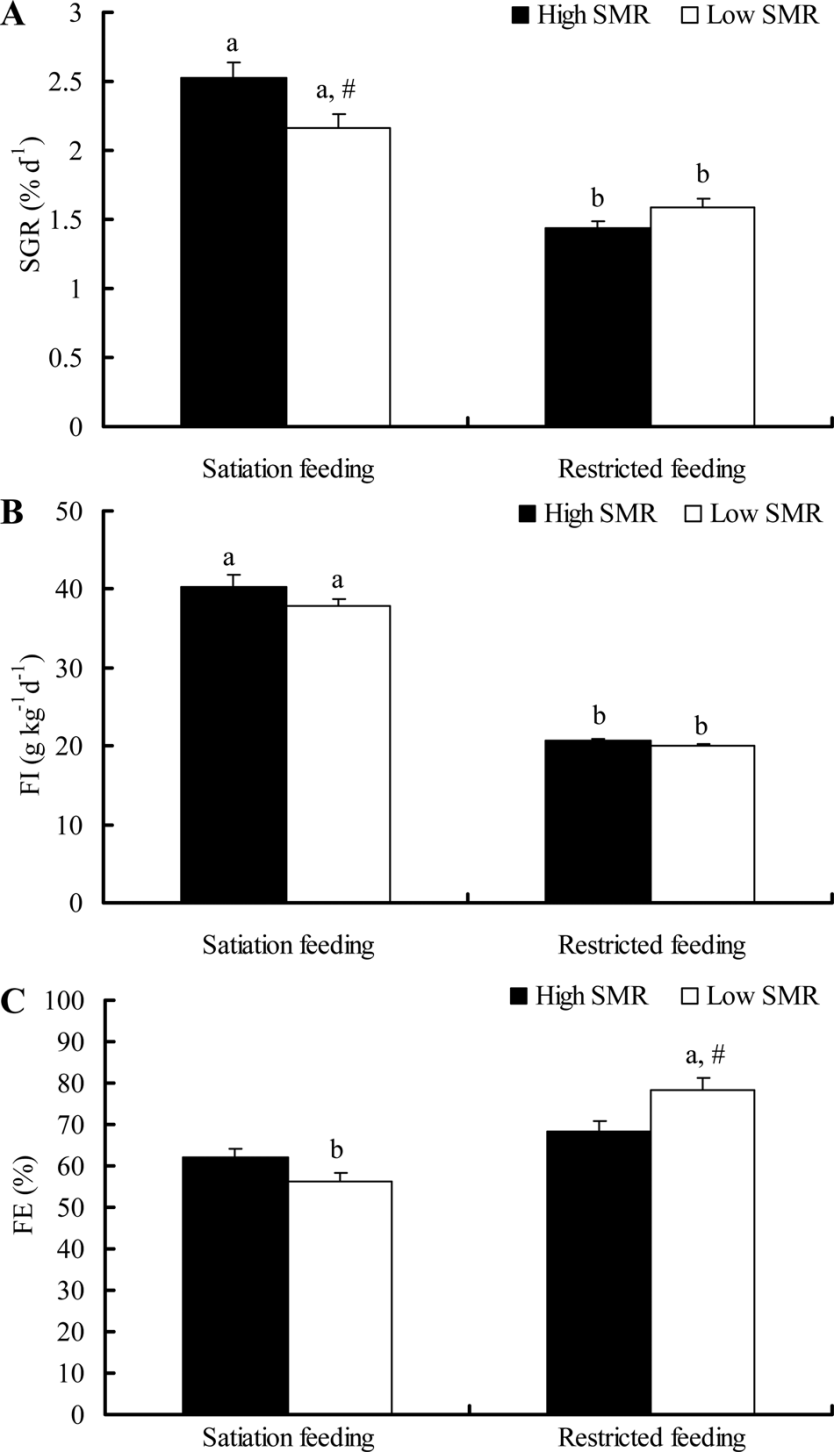
**The SGR, FI and FE of individuals with two SMR phenotypes during feeding.** The sample sizes were 19, 16, 20 and 16 for the high SMR satiation feeding group, low SMR satiation feeding group, high SMR restricted feeding group and low SMR restricted feeding group, respectively. Data are represented as the mean±s.e.m. Note that different common letters (A,B) within a given SMR phenotype are significant differences between two feeding treatments (*P*<0.05). The # symbol indicates a significant difference between high and low SMR phenotype under either satiation or restricted feeding conditions (*P*<0.05). The linear mixed models included SGR, FI and FE as the dependent variables, SMR and food treatment as explanatory variables followed by an independent-samples *t*-test.

**Fig. 2.**
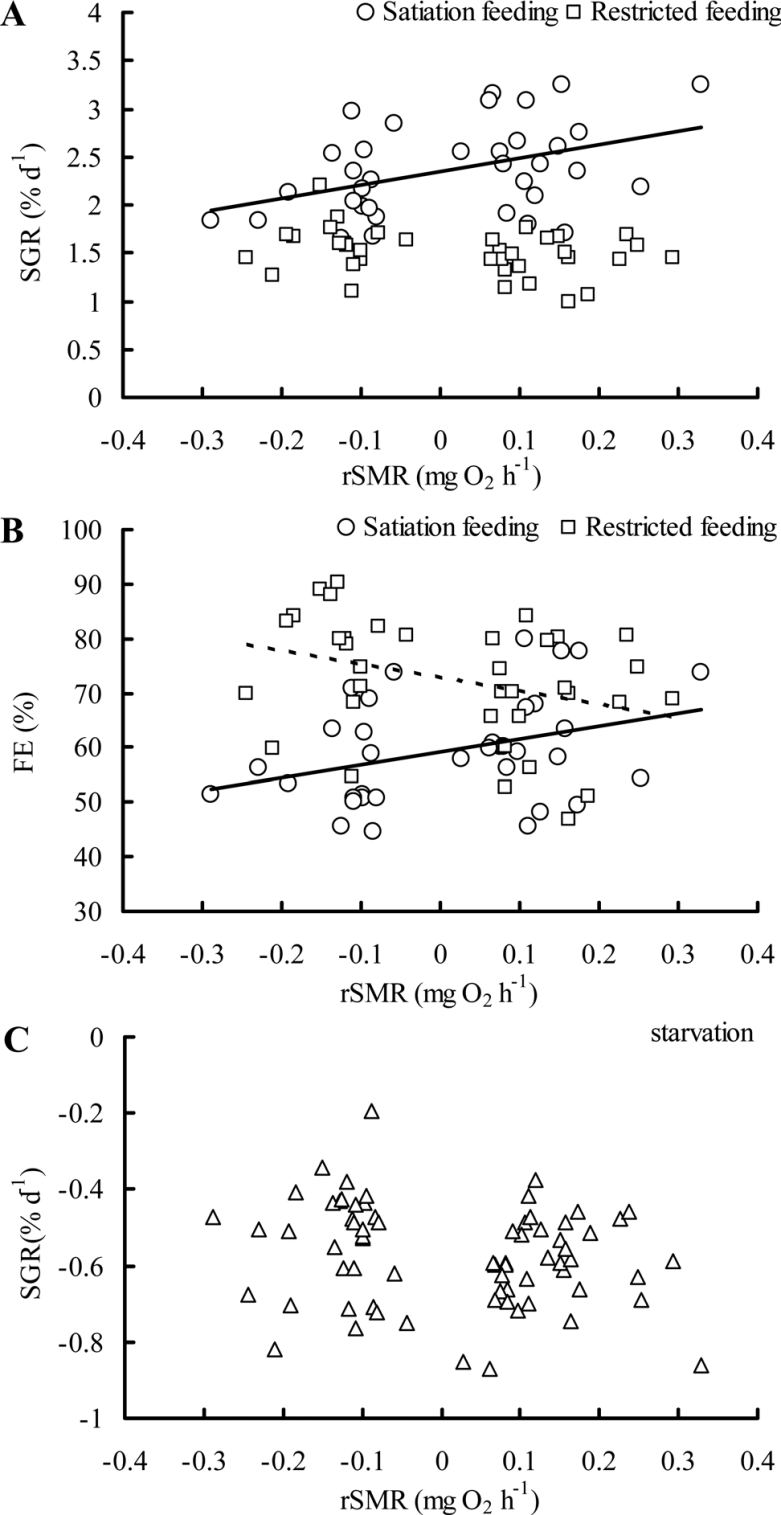
**Correlations between rSMR and growth performance during either feeding or starvation in juvenile crucian carp.** Pearson product-moment correlations are as follows: (A) SGR_satiation feeding_: *r*=0.417, *P*=0.013, *n*=35; SGR_restricted feeding_: *r*=−0.288, *P*=0.088, *n*=36; (B) FE_satiation feeding_: *r*=0.352, *P*=0.038, *n*=35; FE_restricted feeding_: *r*=−0.349, *P*=0.037, *n*=36; (C) SGR_starvation_: *r*=−0.205, *P*=0.085, *n*=71. Solid line (satiation feeding) indicates SGR or FE versus rSMR; dashed line (restricted feeding) indicates FE versus rSMR.

During the feeding phase, specific growth rate (SGR) was higher in the HS group than the LS group (high SMR individuals with satiation food, HS group; low SMR individuals with satiation food, LS group) but did not differ between the HR and LR group (high SMR individuals with restricted food, HR group; low SMR individuals with restricted food, LR group) (Fig. 1A, food: *F*=99.012, *P*<0.001; SMR: *F*=1.451, *P*=0.233; food×SMR: *F*=9.594, *P*=0.003; batch: *F*=3.703, *P*=0.016). A positive relationship among individuals was found between residual SMR (rSMR) and SGR under satiated ration treatment (Fig. 2A, *r*=0.417, *P*=0.013), but not in the restricted ration treatment (Fig. 2A, *r*=−0.288, *P*=0.088). No differences in food intake (FI) were found between high and low SMR individuals within either the satiation group or the restricted group (Fig. 1B, food: *F*=338.93, *P*<0.001; SMR: *F*=1.169, *P*=0.284; food×SMR: *F*=0.709, *P*=0.403; batch: *F*=0.998, *P*=0.400). No differences in feeding efficiency (FE) were detected between the HS and LS group, but the LR group had a larger FE (78.5%) than the HR group (56.4%) (Fig. 1C, food: *F*=37.762, *P*<0.001; SMR: *F*=1.574, *P*=0.214; food×SMR: *F*=7.684, *P*=0.007; batch: *F*=2.055, *P*=0.115). Additionally, a positive and negative relationship was found between rSMR and FE in the satiation group and restricted group, respectively (Fig. 2B, both *P*<0.05).

**Fig. 3.**
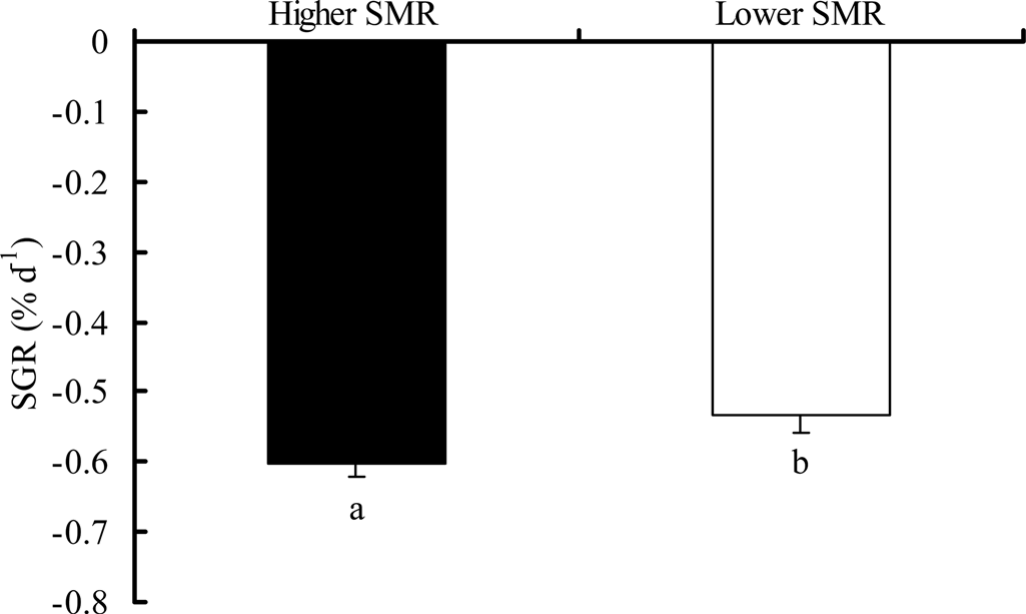
**The decreased characteristics in body mass of the crucian carp with different SMR phenotypes during fasting.** The high SMR group had a greater rate of mass loss during food deprivation as compared to the lower SMR group (two-tailed *t*-test, *t*=2.191, *P*=0.032). Data are represented as the mean±s.e.m. Different common letters indicate significant differences between two SMR phenotypes (*P*<0.05).

The high SMR group had a greater rate of mass loss during food deprivation as compared to the lower SMR group (Fig. 3, *t*-test, *T*=2.191, *P*=0.032). Among individuals, however, rSMR did not correlate with mass loss during food-deprivation (Fig. 2C, *r*=−0.206, *P*=0.085).

## DISCUSSION

The present study found that under conditions of high food availability, individuals with a relatively high SMR had a higher growth rate as compared to those with a low SMR, which is consistent with previous studies (Cutts et al., 1998; Reid et al., 2012; Auer et al., 2015a,b). The improved growth performance of high SMR fish was attributed to a 6.5% increase in FI and 9.8% increase in FE compared to low SMR fish when both groups were fed to satiation. However, such a growth advantage did not exist for high SMR individuals under a restricted diet. Instead, low SMR individuals displayed increased growth rates in this context. This may be partly due to the improved FE of low SMR individuals in the restricted ration treatment. Millidine et al. (2009) found that high SMR individuals of Atlantic salmon (*Salmo salar*) had a higher food processing capacity compared with low SMR individuals during specific dynamic action (SDA) as indicated by a larger magnitude of the total energetic capacity for the ingestion, digestion, absorption and assimilation of a meal (Secor, 2009), and SDA magnitude is generally positively correlated to growth rate (Claireaux and Lefrancois, 2007). Thus, in our study, the decreased FE of low SMR individuals in the satiation treatment may be due to low digestive and assimilative capacities when food is eaten to excess. On the other hand, the higher FE of low SMR individuals under the restricted food condition may be due to their low maintenance metabolism (Burton et al., 2011): nutrients from a given meal are more likely to be allocated to the somatic growth rather than idling costs in low SMR individuals. However, this difference in FE in the restricted ration treatment was offset by the high food processing capacity in the high SMR individuals, especially with the small proportion of energy expenditure on maintenance metabolism relative to ingested energy in the satiation treatment.

During food deprivation, the rate of mass loss in high SMR individuals was greater than that of low SMR individuals, indicating that an individual with relatively low SMR possesses a higher starvation tolerance. Similarly, previous studies on juvenile European sea bass (*Dicentrarchus labrax*) (Dupont-Prinet et al., 2010; Killen et al., 2011; McKenzie et al., 2014), goldfish (*Carassius auratus*) (Liu et al., 2016) and brown trout (*Salmo trutta*) (Auer et al., 2016) have observed that individuals with a higher SMR lose body mass more rapidly during a period of food deprivation. High SMR phenotypes may mobilize internal energy stores (e.g. lipid and protein) more quickly during food deprivation as compared to the more tolerant low SMR phenotypes (McKenzie et al., 2014). It is noteworthy that the observed differences in mass loss were observed at the group level (high versus low SMR) under food deprivation, but not among individuals (there was no correlation among individuals with SMR and mass loss, Fig. 2C). This phenomenon could be due to individual differences in the specific substrates used to fuel metabolism, or differences in the starting amount of whole-body lipid (Auer et al., 2016). Differences in spontaneous activity among individuals throughout the period of food deprivation could also obscure the ability to detect among-individual relationships between mass loss and SMR.

In summary, SMR appears to predict growth performance in juvenile *C*. *auratus* under changing food availability, highlighting the context-dependent advantages of variation in SMR among individuals. Since SMR is a key physiological trait which is often correlated with other phenotypic traits (Burton et al., 2011; Auer et al., 2015a), any context-dependent benefits of SMR may lead to correlated natural selection on these other phenotypic traits (e.g. aerobic capacity) in fish (Auer et al., 2017). Furthermore, the varying costs and benefits of a high or low metabolic demand may help explain how variation in metabolic rates can persist within populations. It should be noted, however, the SMR can exhibit flexibility in response to environmental factors such as food availability, but the ecological consequences of this plasticity are still largely unknown (Van Leeuwen et al., 2012; Norin et al., 2015). Further studies are needed to evaluate the factors that induce this plasticity, the timescales over which plasticity occurs, and the degree to which such adjustments to metabolic traits may help buffer individuals against the negative effects of environmental change.

## MATERIALS AND METHODS

### Animals

Juvenile crucian carp (body mass=8.44±0.13 g, body length=6.81±0.03 cm, *n*=155) were obtained from local fisheries in Chongqing, China. The fish were kept in three recirculating water tanks (length×width×height=1.2 m×0.6 m×0.6 m) for 4 weeks before the experiment, with a 12 h light:12 h dark cycle. The water temperature was maintained at 20.0±0.5°C, and the oxygen content was kept above 7.0 mg l^−1^ by using an air pump. Fish were fed to satiation once daily with Cyprinid fish diet (composition: 41.2±0.9% protein, 8.5±0.5% lipid, 25.7±1.2% carbohydrate, and 12.3±0.4% ash) (Tongwei Ltd, Sichuan, China), which was used for the entire experiment. This fish diet was made in the form of a sphere and could be natant on the surface of the water without dissolving for 12 h. Although there is a slight difference in the diameter of the commercial fish diet, all of the diet pellets were filtrated by using a screen mesh to acquire the same size of pellet (mean mass=0.016 g per pellet) before the experiment. All animal handling and experiments were conducted in accordance with the ethical requirements and recommendations for animal care of the Key Laboratory of Animal Biology of Chongqing, China (Permit No. Zhao-20130125-01) and requirements of environmental and housing facilities for laboratory animals in China (GB/T14925-2001). All the experiments were also complied with the local animal welfare laws of Chongqing City, China.

### Experimental protocol

**Fig. 4.**
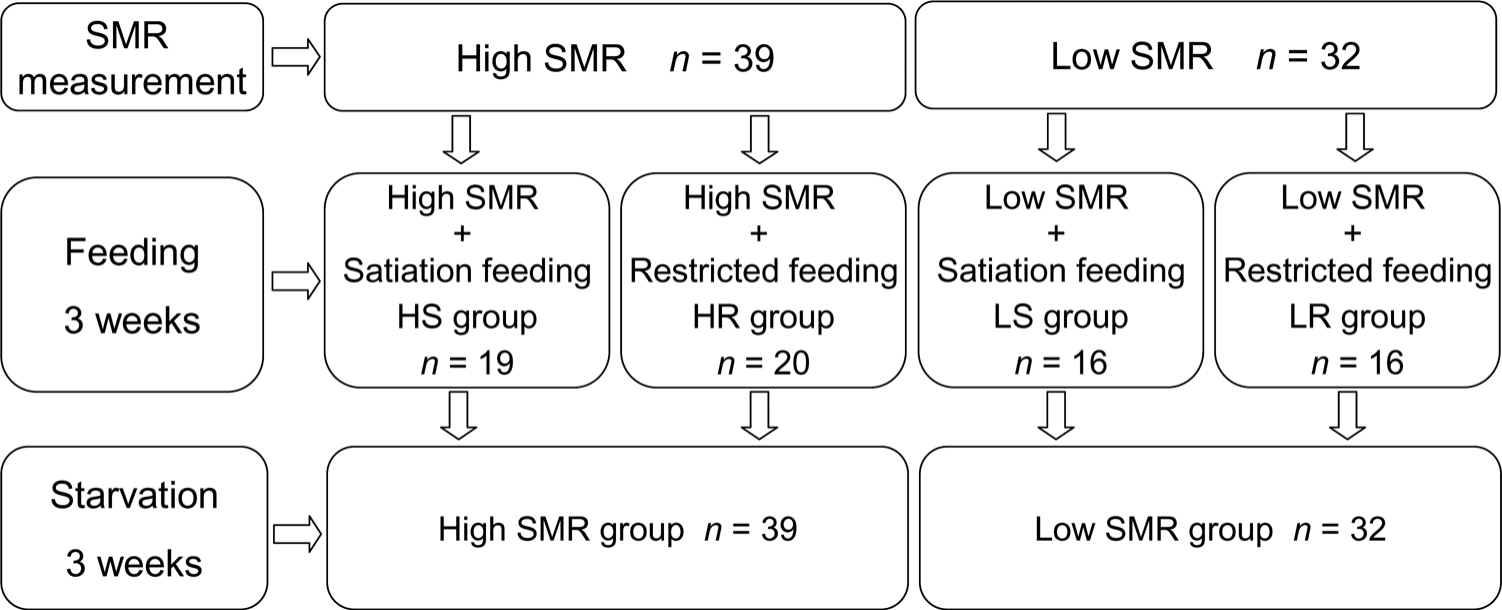
**The flowchart of the present study showing the sequences of food and starvation treatment.** The ration of restricted feeding was 50% of the satiation feeding.

After the 4 week acclimation period, SMR of all fish was estimated by measuring their oxygen uptake after 48 h of fasting (Table 1). After being measured for SMR (see ‘Measurement of SMR’ section) individual fish were classified as having either a high or low SMR (again see ‘Measurement of SMR’ for description of this procedure). The 39 highest and 32 lowest SMR fish out of the 155 fish measured were then further sub-divided randomly to receive either a satiation diet with average 2.0% body mass of fish (high SMR individuals with satiation food, HS group; low SMR individuals with satiation food, LS group) or restricted diet (50% of satiation, high SMR individuals with restricted food, HR group; low SMR individuals with restricted food, LR group) for three weeks (see in Fig. 4; Table S1). To avoid the influence of dominance hierarchies and competition for food, fish were individually raised in 80 separated compartments (length×width×height=17×10×15 cm) in two independent cycling tank systems, which consisted of a large tank (length×width×height=1.1 m×1.1 m×0.8 m, volume=968 l) and a water filtration system.

**Table 1.**
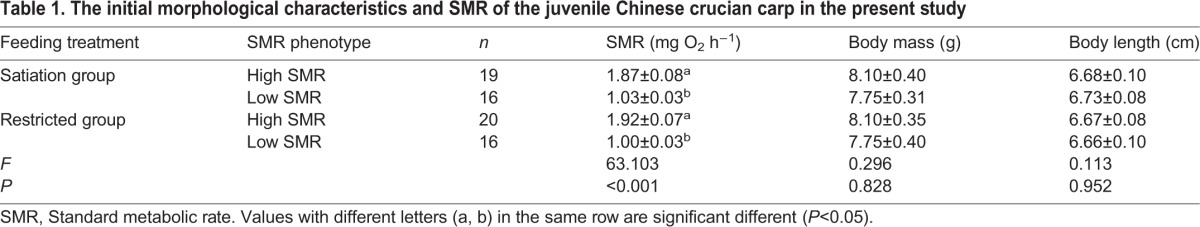
**The initial morphological characteristics** **and SMR of the juvenile Chinese crucian carp in the present study**

After 3 weeks of the satiation and restricted feeding treatments, all experimental fish (*n*=71) were placed on a satiation diet twice daily for five days to reduce differences in nutrient status between SMR groups. All fish were then food deprived for the following 3 weeks. During this food-deprivation period, the HS group and HR group were combined to form the high SMR group, while the LS group and LR group were combined to form the low SMR group on the assumption that the relative SMR of individual fish was maintained despite the recent dietary differences between treatments. Each day, 10% of the water in the cycling tank system was replaced by dechlorinated tap water. All environmental conditions (e.g. temperature, oxygen concentration) of the cycling tank system were the same as during the acclimation period.

### Growth measurements

Fish were fed twice daily (9:00 h and 19:00 h) after they were transferred into the individual compartments. During each feeding, fish in the satiation treatment were fed to excess and uneaten pellets were collected and counted to calculate the feed intake. The restricted ration treatment was fed one half of the mean diet pellets of the satiation group. Fish from the restricted group consumed all food provided during each feeding period. One hour after feeding, feces were removed from all compartments using a siphon. Body mass (to the nearest 0.01 g) and body length (to the nearest 0.1 cm) of individual fish were measured after the fish were slightly anesthetized with buffered tricaine methane sulphonate (MS-222, 50 mg l^−1^) at the end of each week during both the feeding and food-deprivation period.

The following formulas were used to calculate the parameters of feeding and growth:
(1)
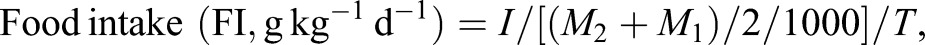

(2)
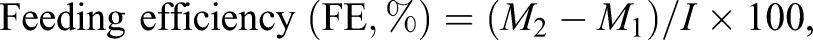

(3)
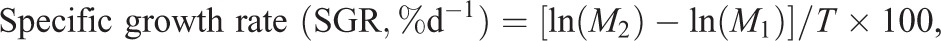
where *M*_1_ and *M*_2_ are the body mass (g) of fish at the beginning and end of a specific period (21 days), respectively, while *I* (g) represents the total amount of diet ingested for a specific period, and *T* denotes the duration (21 days).

### Respirometry and measurement of SMR

SMR was estimated at the beginning of the experiment by measuring the rate of oxygen uptake using four sets of continuous-flow respirometers, each comprising 10 separate fish chambers (24.0 cm length×5.0 cm diameter) and one control chamber (without fish) at 20.0±0.5°C (Fu et al., 2009). For each respirometer, the 11 chambers were arranged in parallel and submerged in a transparent water bath. Under the bath there was a large reservoir (length×width×height=0.8 m×0.8 m×0.55 m) in which the oxygen content of the water was kept to saturation using an air pump. A submerged pump moved water from the reservoir to a small water tower above the bath tank to generate a constant flow through the respirometry chambers. A flow-control switch was located between the tower and a distributing pipe to each chamber with an average flow fate of 3.0 l h^−1^. Water from the outlet of each chamber passed through a biological filter and then returned to the reservoir tank.

To avoid the influence of digestion on measurements of SMR, fish were fasted for 48 h prior to being placed into respirometry chambers, by which time fish had evacuated their guts (Fu et al., 2009; Li et al., 2015). The following day, oxygen uptake was measured at three time points (10:00 h, 15:00 h, and 20:00 h) and repeated three times at each time point. Repeatability across time periods was high [intraclass correlation coefficient (ICC)=0.840, *P*<0.001], and the three measurements were averaged to provide a value for SMR. The oxygen concentrations of the outlet of the chambers were measured by using an oxygen meter (HQ_30_, Hach Company, Loveland, CO, USA) and remained above 80% saturation. The flow rate (average 3.0 l h^−1^) was determined by measuring the time taken for filling the volumetric flask of 100 ml from outlet of the respirometry chambers. In the present study, SMR of the individual fish was determined by measuring the oxygen consumption rate (*M*O_2_, mg O_2_ h^–1^), which was calculated as follows:
(4)
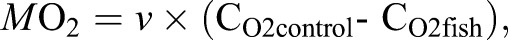
where *v* (l h^–1^) is the flow rate of water through the respirometer, average time for up to 99% water transformation in chamber is less than 28 min under the flow rate of 3.0 l h^−1^. C_O2control_ and C_O2fish_ ΔO_2_ are the oxygen concentrations (mg l^−1^) in the outflow water of control chamber and fish respirometer, respectively.

Before classification for the high and low SMR groups, the relationship between log SMR and log body mass was examined because body mass has a strong affect on absolute and mass-specific metabolic rates. Indeed, such relationship was found (intercept: −0.064±0.100, β: 0.223±0.108, *P*=0.041, *R*^2^=0.027, *n*=155). The rSMR for individual fish from the regression was determined according to whether it had a positive or a negative rSMR, and then each fish was categorized as high or low SMR for a given body mass (Cutts et al., 1998). Based on the results of regression, the present study used 39 high SMR individuals and 32 low SMR individuals for the experiment according to the rank of rSMR, and the remainder (*n*=84) was not employed for the experiment due to insufficient cultured compartments.

### Data handling and analysis

Linear mixed effect models were used to analyze the data. The models included SGR, FI and FE as the dependent variables, SMR and food treatment as explanatory variables. Because fish were measured for SMR in batches (*n*=40 fish per group), respirometry batch number was included as a random effect in the analyses. When there were differences between food treatments or SMR groups, we performed a post hoc LSD multiple comparison. The initial morphological characteristics and SMR among different SMR phenotypes was performed by one-way ANOVA followed by an LSD test. An independent-samples *t*-test was used to detect differences in SGR value between the high SMR group and the low SMR group during food deprivation. Pearson correlation was used to test the relationship between rSMR and SGR and FE. All analyses were performed using SPSS v19.0 (SPSS Inc. and IBM, Chicago, USA). *P*<0.05 were considered statistically significant, and all data are presented as the means±s.e.m.
